# CT angiography–derived plaque and perivascular fat radiomics for predicting ipsilateral stroke recurrence in patients with carotid atherosclerosis

**DOI:** 10.3389/fneur.2026.1821860

**Published:** 2026-04-16

**Authors:** Jianyong Wei, Yulu Yang, Xiaoer Wei, Zhiwen Yang, Jingying Tao, Chao Zheng, Shundong Hu, Yueqi Zhu, Yuehua Li

**Affiliations:** 1 School of Health Science and Engineering, University of Shanghai for Science and Technology, Shanghai, China; 2 Institute of Diagnostic and Interventional Radiology, Shanghai Sixth People’s Hospital Affiliated to Shanghai Jiao Tong University School of Medicine, Shanghai, China; 3 ShuKun Technology Co., Ltd., Beijing, China

**Keywords:** carotid plaques, computed tomography angiography, perivascular adipose tissue, radiomics, stroke recurrence

## Abstract

**Objective:**

To develop and validate an interpretable machine learning (ML) model integrating computed tomography angiography (CTA)-derived radiomics features of carotid plaque and perivascular adipose tissue (PVAT) for predicting ipsilateral stroke recurrence in patients with carotid atherosclerosis.

**Methods:**

In this retrospective study, patients with unilateral carotid atherosclerosis who underwent head and neck CTA between May 2016 and March 2024 were included and followed for recurrent ischemic stroke detected by follow-up MRI. Radiomics features of carotid plaque and PVAT were automatically extracted using a deep learning-based segmentation model and then gathered to constructed a ML model to predict stroke risk. A conventional clinical model based on carotid stenosis degree and clinical factors was also developed. Additionally, a combined model incorporating both radiomics and clinical factors was constructed. The optimal predictive model was chosen among five ML algorithms based on the area under receiver operating characteristics curve (AUC). Model performance was validated through repeated 10-fold cross-validation and tested in an independent testing cohort. Model interpretability was examined using Shapley Additive Explanations (SHAP).

**Results:**

Of 162 patients (mean age, 69.28 years ± 8.30 [SD]; 136 [83.95%] male) were included, of whom 63 (38.9%) experienced ipsilateral stroke recurrence during follow-up (median, 1 years). The combined model using support vector machines achieved the highest AUC of 0.87(95% CI: 0.74–0.97) in the testing set, higher than the radiomics-only model (AUC, 0.80; 0.63–0.94) and the clinical model (AUC, 0.77; 0.58–0.91; all *p* < 0.05). SHAP analysis demonstrated that plaque texture features contributed strongly to recurrence risk, while PVAT-derived features provided complementary inflammatory information.

**Conclusion:**

An ML model including both radiomics features of carotid plaque and PVAT can improve performance in predicting ipsilateral stroke recurrence risk than clinical factors alone, offering a promising tool for stroke prevent in clinical practice.

## 1 Introduction

Stroke remains a leading cause of mortality and long-term disability worldwide, and carotid atherosclerosis (CAS) accounts for a substantial proportion of ischemic cerebrovascular events (1, 2). Patients with CAS who have experienced prior ischemic events remain at substantial risk of subsequent ipsilateral stroke, particularly within the first years after the index event (3, 4). Accurate risk stratification in this population is essential to guide intensified medical management and identify individuals who may benefit from closer surveillance or revascularization (5). However, current clinical decision-making is largely guided by luminal stenosis severity, which incompletely reflects plaque vulnerability and limited to fully capture the complex biological processes underlying recurrent cerebrovascular events (6–8). This highlights an urgent need for refined imaging biomarkers capable of identifying high-risk CAS patients beyond luminal narrowing.

Advanced vascular imaging has improved the characterization of carotid plaque morphology and composition. High-resolution magnetic resonance imaging (MRI) has demonstrated that intraplaque hemorrhage, lipid-rich necrotic core, and fibrous cap disruption are associated with future cerebrovascular events (9, 10). However, MRI remains limited by cost, accessibility, and workflow constraints in routine clinical practice (11). In contrast, computed tomography angiography (CTA) is widely available and routinely performed in cerebrovascular evaluation (12, 13). Previous studies have demonstrated that CTA can provide valuable information about plaque composition, calcification patterns, and remodeling features that correlate with plaque instability and stroke risk (8, 14, 15). Recent machine learning (ML) studies using CTA-derived plaque characteristics have shown promise in identifying symptomatic plaques (8, 16).

Beyond plaque morphology, increasing attention has been directed toward perivascular adipose tissue (PVAT) as a dynamic regulator of vascular inflammation. Experimental and clinical evidence supports bidirectional inflammatory signaling between the arterial wall and adjacent adipose tissue. In coronary artery disease, CT-derived PVAT has been validated as a noninvasive biomarker of vascular inflammation and residual cardiovascular risk (17, 18). Extending this concept to carotid disease, several studies have shown that increased PVAT and plaque features are associated with symptomatic plaques and imaging markers of vulnerability (19, 20), and radiomics-based PVAT models achieved moderate discriminative performance in differentiating symptomatic from asymptomatic plaques (21–23). However, most carotid PVAT investigations have relied on two-dimensional attenuation measurements or manually defined regions of interest, potentially overlooking the complex three-dimensional heterogeneity and spatial interaction between plaque structure and its surrounding inflammatory microenvironment. However, whether quantitative PVAT imaging features provide incremental value for predicting recurrent stroke remains insufficiently explored.

Radiomics provides a high-dimensional quantitative framework capable of capturing spatial texture patterns and intensity distributions beyond conventional visual interpretation (24–26). By characterizing tissue heterogeneity at the voxel level, radiomics may better reflect underlying plaque composition and inflammatory remodeling. Prior studies have applied radiomics to either plaque or PVAT to differentiate symptomatic from asymptomatic lesions, demonstrating moderate diagnostic performance (27–31). However, integration of plaque and PVAT radiomic features within a fully automated three-dimensional pipeline for individualized stroke risk prediction remains insufficiently explored. Therefore, we hypothesized that quantitative radiomic features derived from both carotid plaque and adjacent PVAT on CTA could provide synergistic information for predicting ipsilateral recurrent stroke. In addition to making predictions based on unseen data, ML models can enhance interpretability through Shapley Additive Explanations (SHAP) (32). This effectively reveals the specific risk factors and their degrees of impact on stroke occurrence.

Therefore, this study aimed to develop and validate a fully automated CTA-based plaque and PVAT radiomics model integrating carotid plaque and PVAT features to predict ipsilateral stroke recurrence in patients with CAS. We further evaluated whether combining radiomics with clinical factors improves predictive performance and applied SHAP to enhance model interpretability. By shifting from cross-sectional plaque characterization to individualized recurrence risk prediction, this approach seeks to refine secondary prevention strategies in carotid artery disease.

## 2 Materials and methods

### 2.1 Patient selection

This study protocol was approved by the ethics committee of Shanghai Jiao Tong University Affiliated Sixth People’s Hospital (No. 2024-KY-097(K)). The requirement for obtaining written informed consent was waived owing to the use of de-identified imaging data.

**Figure 1:**
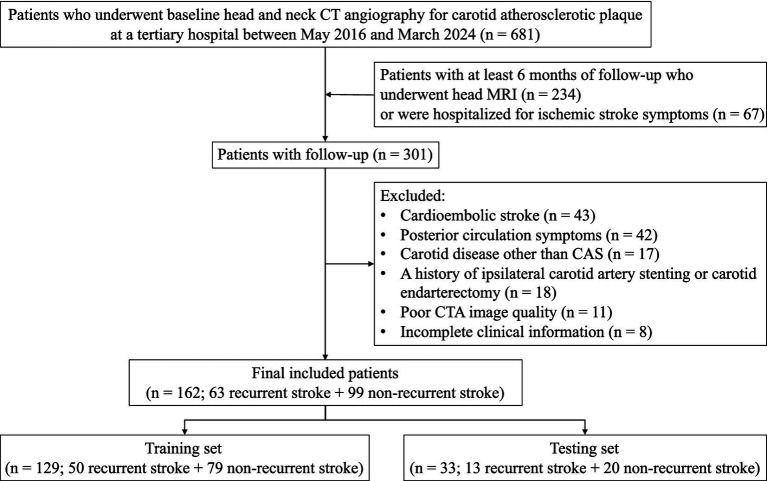
Flowchart of patient selection. CAS, carotid atherosclerosis; CTA, computed tomography angiography; MRI, magnetic resonance imaging.

This study retrospectively enrolled 681 patients with extracranial CAS plaques who underwent head and neck CTA at Shanghai Jiao Tong University Affiliated Sixth People’s Hospital between May 2016 and March 2024. The inclusion criteria were as follows: (1) Age ≥ 18 years; (2) Unilateral extracranial carotid atherosclerotic plaque detected on CTA; (3) A documented history of ipsilateral ischemic stroke or transient ischemic attack (TIA) within 6 months prior to the index CTA examination (33); (4) At least 6-months follow-up with head MRI examination, or hospitalization due to ischemic stroke symptoms. The exclusion criteria were: (1) Cardioembolic stroke confirmed by TOAST (*n* = 43); (2) Posterior circulation stroke, or in case of doubt with other pathologies (hypoglycemia, migraine, post paroxysmal neurological dysfunction) (*n* = 42); (3) Carotid disease other than CAS, such as carotid dissection, aneurysm, primary intracranial disease, or radiation therapy (*n* = 17); (4) A history of ipsilateral carotid artery stenting or carotid endarterectomy (*n* = 18); (5) Poor CTA image quality precluding analysis (*n* = 11); (6) Incomplete clinical information (*n* = 8). Ultimately, 162 patients were included and randomly divided into training set (*n* = 129) and testing set (*n* = 33), corresponding to an approximate 8:2 split (Figure 1). Baseline demographic and vascular risk factors were detailed in Appendix E1.

### 2.2 Clinical outcome definition

The primary endpoint of this study was ipsilateral recurrent ischemic stroke during follow-up. All patients were divided into recurrent stroke or non-recurrent stroke groups based on the diagnosis according to clinical symptoms, time of onset, and imaging data by an experience neurologist (X.E.W., over 15 years’ experience). Recurrent stroke was defined as a new neurological deficit with a National Institutes of Health Stroke Scale (NIHSS) score ≥2 accompanied by a new diffusion-weighted imaging (DWI) lesion ≥1.5 cm on MRI, occurring after the index event (34, 35). To ensure that the recurrence was attributable to the index carotid atherosclerotic lesion, the recurrent infarction was required to occur within the vascular territory of the ipsilateral carotid artery corresponding to the baseline plaque location. Transient ischemic attacks without imaging evidence of infarction were not considered recurrence events in this study. This definition was adopted to improve diagnostic specificity and reduce the potential misclassification associated with transient neurological symptoms without radiographic confirmation. Patients without any new ipsilateral ischemic events during the follow-up period were classified as the non-recurrence group.

### 2.3 CTA acquisition protocol

CTA scans were performed using scanners from four different manufacturers (Brilliance ICT, Philips; Somatom Sensation, Siemens; UIH; and GE Healthcare). Specific CT scanners and parameters are detailed in Supplementary Table S1. Patients were positioned supine with calm breathing, and the scanning range extended from the aortic arch to the cranial vertex. A 50 mL dose of iodine contrast agent was injected at a rate of 4.0 to 5.0 mL/s. The scan was triggered 3 to 4 s after a delay once the attenuation threshold in the region of interest (the aortic arch at the level of the carina) reached 100 HU. Scanning parameters were as follows: tube voltage of 100 kV; automatic milliampere control; matrix size of 512 × 512; field of view of 250 × 250 mm; slice thickness of 0.5 to 1 mm; and slice spacing of 0.5 mm. MRI scan parameters at the 6-month follow-up were shown in Supplementary Table S2.

### 2.4 Plaque and PVAT segmentation

**Figure 2:**
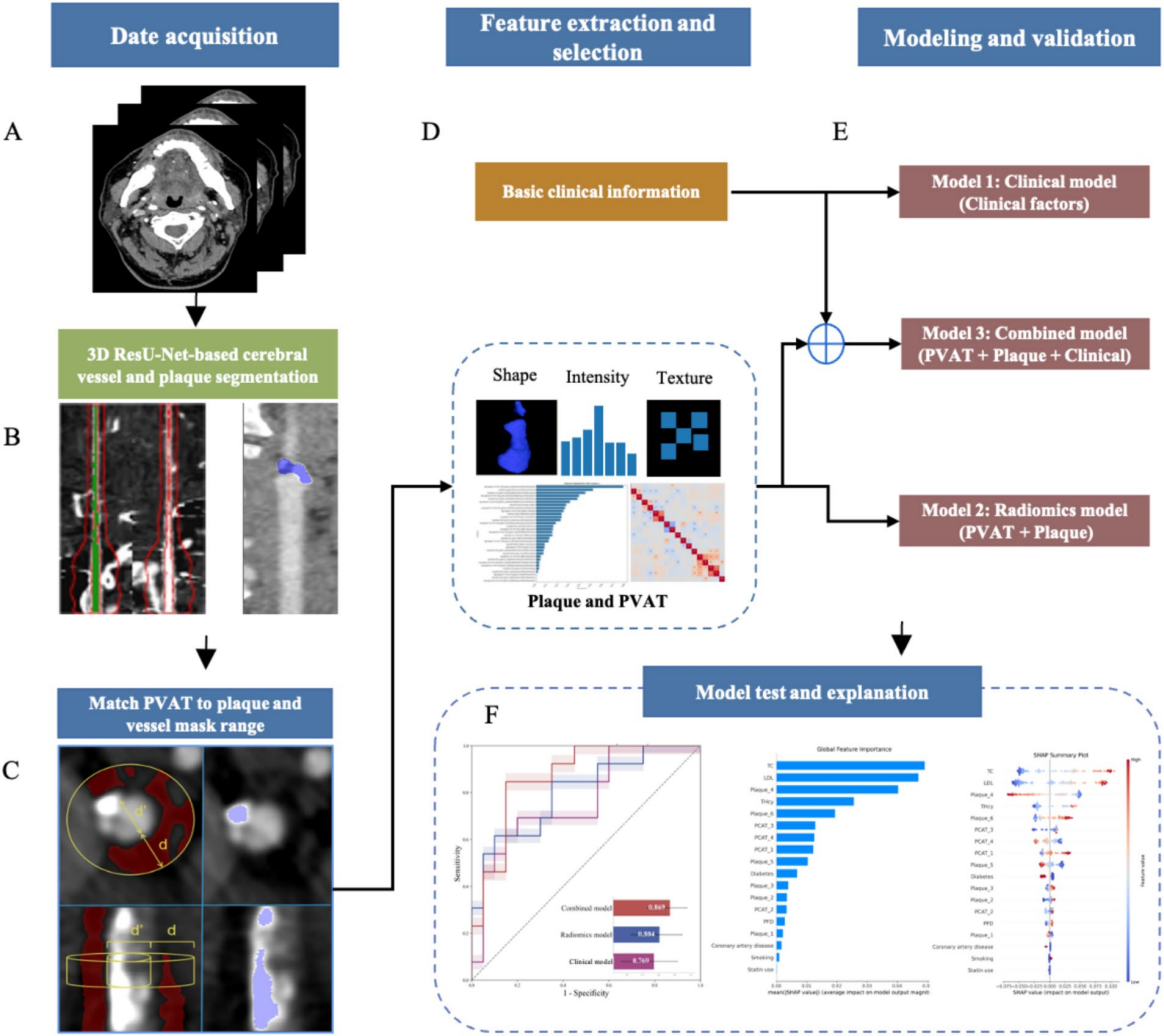
Comprehensive workflow for radiomics analysis and model development. **(A)** Acquisition of head and neck computed tomography angiography (CTA) images. **(B)** Automated vessel segmentation was performed using a 3D ResU-Net, followed by manual plaque annotation by experienced radiologists. **(C)** Perivascular adipose tissue (PVAT) mapping: the PVAT region was defined and spatially aligned with the corresponding plaque and vessel mask range. **(D)** Radiomics features were extracted from both plaque and PVAT regions, and feature selection was conducted to identify the most predictive variables. **(E)** Model development and validation: three machine learning–based predictive models were constructed. **(F)** Model performance was evaluated in the testing cohort, and interpretability was assessed using Shapley additive explanations (SHAP) to visualize feature contributions.

The core design of the fully automated plaque and PVAT segmentation was based on the CerebralDoc system, previously developed by the author team using deep-learning based models (36–38). To reduce scanner-related variability, all original images were preprocessed, isotopically resampled to a voxel spacing of 1 × 1 × 1 mm^3^, and intensity-normalized using Z-score normalization prior to radiomics feature extraction. The segmentation process was descripted in Figure 2 and carried out as follows: (1) The optimized 3D ResU-Net model was primarily applied to segment the head and neck vessel (38); (2) Based on the vessel segmentation, a weighted skeleton method, combined with the topology of prior knowledge, was used to extract the most accurate vessel centerline (37); (3) Using the obtained centerline, a straightened rendering of the artery was generated, which enabled precise visualization the vessel lumen and plaque; (4) A 3D ResU-Net model, combined with a multi-feature sequence callback model, was then applied to both the original axial and straightened images for plaque segmentation (36); (5) For the calculation of PVAT near the plaque (39): Based on the vessel and plaque segmentation results from the straightened images, the centerline was used to compute the area of each vessel cross-section and the theoretical lumen radius. A circular contour with a radius 1.5 times the lumen radius was drawn around the vessel center to define the perivascular region, based on previous studies evaluating PVAT surrounding vascular structures (21, 23). This approach ensures consistent capture of the perivascular adipose tissue immediately adjacent to the vessel wall while minimizing inclusion of distant fat unrelated to vascular inflammation. Using the original vessel straightened images, a threshold range of −30 HU to −190 HU was applied for PVAT segmentation. A thresholding algorithm was used to obtain the fat mask surrounding the vessel, which was then intersected with the circular contour to yield an initial fat segmentation mask. Finally, post-processing methods, such as removing isolated fatty points based on large connected domain volume, were applied to refine the mask and obtain the final fat segmentation. The PVAT around the plaque was calculated as the mean CT Hu value of the fat segmentation mask within the plaque region on the straightened image. Based on automated segmentation pipeline, PVAT mask, plague mask and vascular lumen stenosis were recorded. The degree of stenosis was measured on the original CTA images according to the North American Symptomatic Carotid Endarterectomy Trial (NASCET) criteria, with stenosis classified into three grades: mild (<50%), moderate (50–69%), and severe (70–99%) (40).

### 2.5 Radiomics feature extraction and selection

Radiomic features were extracted from both plaque and PVAT regions using (version 3.7; https://github.com/Radiomics/pyradiomics). Before extracting the radiomic features, all CTA images underwent preprocessing. First, gray level discretization was performed using a fixed bin width of 25. Then, B-spline interpolation was applied to resample the images to a uniform resolution with a voxel size of 1 × 1 × 1 mm^3^. Subsequently, Z-score normalization ( Z=[X−μ]/σ , where X is the pixel intensity value, μ is the mean pixel intensity, and σ is the standard deviation of pixel intensities) was used to normalize the images, and outliers were removed during this process (41). This normalization step partially mitigated the heterogeneity of data originating from different centers and types of equipment. Following normalization, image processing was conducted using a combination of Laplacian of Gaussian filters with kernel sizes of 0.5, 1, 1.5, and 2, along with wavelet transformations.

Feature selection was performed exclusively in the training cohort. The optimal radiomic features were selected according to the ranked from the highest-to-lowest importance. Firstly, the Shapiro–Wilk test was applied to assess the distribution of each feature. Depending on the distribution, either an independent samples *t*-test or Mann–Whitney *U* test was used to identify features with significant differences between groups (*p* < 0.05). Secondly, Least Absolute Shrinkage and Selection Operator (LASSO) with 10-fold cross-validation was employed to select the optimal regularization parameter and further refine the feature set. Finally, The Pearson correlation coefficient was calculated to identify and eliminate highly collinear features (|r| > 0.9). When two radiomic features exhibited collinearity, the feature with the smaller *p*-value was retained.

### 2.6 Model development, evaluation and interpretation

The study workflow is illustrated in Figure 2. In this study, the dataset was randomly divided into a training cohort (*n* = 129) for model development and a testing cohort (*n* = 33) for independent performance evaluation, corresponding to an approximate 8:2 split. The training cohort was used for model development and internal cross-validation, whereas the testing cohort was reserved exclusively for independent performance evaluation. The primary purpose of the training set was to construct ML models, while the testing set was reserved exclusively for assessing the models’ predictive capabilities. This study focused on the application of five prevalent ML algorithms—Decision Tree, Logistic Regression (LR), Random Forest (RF), Boosting (XGBoost), and Support Vector Machine (SVM)—for predicting stroke recurrence. The final selected radiomic features from carotid plaque and PVAT were used to construct the radiomics prediction model. For comparison, a clinical model was built based on basic clinical features that were significant in univariate tests (*p* < 0.05) and vascular stenosis evaluated by an experienced radiologist (X.Y.S, with over 5 years of experience). Additionally, a combined radiomics-clinical model incorporating both radiomic features and basic clinical features was developed. All models were trained for two binary predictions tasks defined according to the two predefined outcomes. To avoid overfitting during model development, 10-fold cross-validation was used for the selection of hyperparameters and model architecture (42). Briefly, the data is divided into 10 equally sized subsets, ensuring similar proportions of cases in each subset. Nine out of 10 sets were used for model derivation and the remaining set was used for validation. This protocol was applied 10 times so that each set was used for validation exactly once. The cross-validation procedure was applied after feature selection on the training data to optimize model hyperparameters and estimate internal performance. The independent testing cohort remained completely separate and was not used in any feature selection or model training steps, ensuring that no information leakage occurred between training and testing datasets. Overall, this scheme was repeated with different data shuffles to better estimate the generalization error of the model. The optimal classification threshold was determined by maximizing Youden’s index to further improve the model’s discriminative ability.

In assessing model performance, metrics included the area under the receiver operating characteristic curve (AUC), accuracy, sensitivity, and specificity. To enhance reliability, 95% confidence intervals (CI) were estimated using 1,000 bootstrap samples. By constructing ROC curves and comparing AUC values, we identified the model with the highest prediction performance. Furthermore, feature ranking evaluation served as a method to quantify the significance of individual features within a dataset, gauging their influence on the final outcome.

For model interpretation, the Shapley additive explanations (SHAP) algorithm (43, 44), derived from coalitional game theory, was used to obtain explanations of how individual features drive patient-specific predictions from the previously obtained model. SHAP identifies the contribution of each feature to the prediction by considering all possible coalitions of features. It then assigns a value to each feature that represents its contribution to the prediction, taking into account the impact of the other features. Positive and negative SHAP values indicate an increment or decrement of the prediction score, respectively (Figure 2).

### 2.7 Statistical analysis

All statistical analyses were conducted using SPSS (version 23.0, IBM Corp.), Python (version 3.11.6, Python Software Foundation), and MedCalc (version 18.2.1, MedCalc Software Ltd.). Categorical variables were expressed as frequencies and percentages, while continuous variables were described as mean ± standard deviation (SD) based on normality. Categorical variables were compared using the *χ*^2^ test, and normality of continuous variables was assessed using the Shapiro–Wilk test. For normally distributed variables, the *t*-test was applied, while the Mann–Whitney *U* test was used for non-normally distributed variables. Significant variables (*p* < 0.05) from traditional basic clinical features were included in the model via multivariate logistic regression analysis. Correlations between traditional and radiomic features were determined using the Pearson correlation coefficient. The predictive performance of each model was compared using the Delong test, with *p* < 0.05 indicating statistical significance. The carotid PVAT measurements were reported in HU.

## 3 Results

### 3.1 Patient characteristics

A total of 162 patients (mean age, 69.3 years ± 8.3 [SD]; 136 men) were included in the final analysis. During follow-up, 63 patients (38.9%) experienced recurrent ipsilateral ischemic stroke, while 99 patients (61.1%) remained recurrence-free. The median time to recurrence was 12 months (interquartile range [IQR], 6–16 months) after the index event. Baseline clinical and imaging characteristics stratified by recurrence status are summarized in Table 1. Detailed comparisons of patient characteristics within the training and testing sets are provided in Supplementary Tables S3 and S4, respectively.

**Table 1:** Baseline characteristics.

Characteristics	Total (*n* = 162)	Non-recurrent (*n* = 99)	Recurrent (*n* = 63)	Statistic	Univariate *p* value	Multivariate	
						OR (95% CI)	*p* value
Demographics							
Age, year	69.28 ± 8.30	69.13 ± 7.06	69.52 ± 9.99	*t* = −0.29	0.770		
BMI, kg/m^2^	23.81 ± 3.21	24.19 ± 2.92	23.26 ± 3.55	*t* = 1.60	0.112		
Sex, *n* (%)				*χ*^2^ = 48.67	0.172		
Female	26 (16.05)	19 (19.19)	7 (11.11)				
Male	136 (83.95)	80 (80.81)	56 (88.89)				
Smoking, *n* (%)	38 (23.46)	18 (18.18)	20 (31.75)	*χ*^2^ = 3.95	0.047	1.95 (0.83–4.56)	0.124
Diabetes, *n* (%)	52 (32.10)	38 (38.38)	14 (22.22)	*χ*^2^ = 4.61	0.032	0.35 (0.15–0.81)	0.014
Hypertension, *n* (%)	112 (69.14)	70 (70.71)	42 (66.67)	*χ*^2^ = 0.29	0.587		
Atrial fibrillation, *n* (%)	8 (4.94)	4 (4.04)	4 (6.35)	*χ*^2^ = 0.08	0.772		
Hyperlipidemia, *n* (%)	5 (3.09)	3 (3.03)	2 (3.17)	*χ*^2^ = 0.00	1		
Coronary artery disease, *n* (%)	25 (15.43)	20 (20.20)	5 (7.94)	*χ*^2^ = 4.44	0.035	0.39 (0.11–1.34)	0.133
Antiplatelet use, *n* (%)	10 (6.17)	8 (8.08)	2 (3.17)	*χ*^2^ = 0.87	0.352		
Statin use, *n* (%)	16 (9.88)	15 (15.15)	1 (1.59)	*χ*^2^ = 7.96	0.005	0.16 (0.02–1.32)	0.089
Antihypertension use, *n* (%)	49 (30.25)	31 (31.31)	18 (28.57)	*χ*^2^ = 0.14	0.711		
Antidiabetic use, *n* (%)	66 (40.74)	38 (38.38)	28 (44.44)	*χ*^2^ = 0.59	0.444		
History of stroke, *n* (%)	47 (29.01)	35 (35.35)	12 (19.05)	*χ*^2^ = 4.97	0.026		
Imaging parameters							
Stenosis, *n* (%)				*χ*^2^ = 0.24	0.888		
<50%	11 (6.79)	6 (6.06)	5 (7.94)				
50–69%	19 (11.73)	12 (12.12)	7 (11.11)				
70–99%	132 (81.48)	81 (81.82)	51 (80.95)				
PVAT, HU	−59.02 ± 10.56	−60.98 ± 10.70	−53.52 ± 13.25	*t* = −2.45	0.029	1.81(1.09–2.51)	0.021
Laboratory tests							
TC	4.31 ± 1.34	3.92 ± 1.05	4.91 ± 1.53	*t* = −4.41	<0.001	1.86 (1.13–3.08)	0.015
TG (mmol/L)	1.43 ± 0.87	1.38 ± 0.93	1.51 ± 0.76	*t* = −0.90	0.370		
HDL (mmol/L)	1.08 ± 0.26	1.09 ± 0.25	1.07 ± 0.28	*t* = 0.53	0.599		
LDL	2.66 ± 1.56	2.39 ± 1.72	3.06 ± 1.19	*t* = −2.65	0.009	1.03 (0.62–1.72)	0.901
THcy (μmol/L)	14.57 ± 7.02	13.53 ± 8.22	15.65 ± 5.34	*t* = −1.70	0.091	1.04 (0.98–1.10)	0.234
Manufacturer, *n* (%)							
GE	40 (24.69)	25 (25.25)	15 (23.81)				
Philips	46 (28.40)	32 (32.32)	14 (22.22)				
SIEMENS	47 (29.01)	40 (40.40)	7 (11.11)				
UIH	29 (17.90)	2 (2.02)	27 (42.86)				

Data are means ± standard deviations (SD) for continuous variables and frequencies and percentages for categorical variables. *t* = *t*-test; *χ*^2^ = Chi-square test. BMI, body mass index; CI, confidence interval; HDL, high-density lipoprotein; LDL, low-density lipoprotein; OR, odds ratio; PVAT, perivascular adipose tissue; TC, total cholesterol; TG, triglycerides; tHcy, serum homocysteine.

### 3.2 Feature selection

A total of 1,070 quantitative radiomics features were extracted from the segmented plaque and PVAT regions, encompassing first-order intensity, shape, texture features, and wavelet-filtered features (details in Supplementary Table S5). Using Lasso-based selection (Supplementary Figure S1), the optimal *λ* values were determined to be 0.0915 for plaque and 0.0635 for PVAT features, a total of 10 features were ultimately selected from the plaque (*n* = 6) and the PVAT radiomics (*n* = 4; detailed in Supplementary Table S6) for radiomics model. The multivariate logistic regression analysis revealed that diabetes, TC, and mean PVAT value were significantly associated with the recurrent stroke group (*p* < 0.05, Table 1). In addition, vascular stenosis and indicators with important clinical significance in the univariate analysis was included in the clinical predicting model.

### 3.3 Model performance in predicting recurrence stroke risk

**Figure 3:**
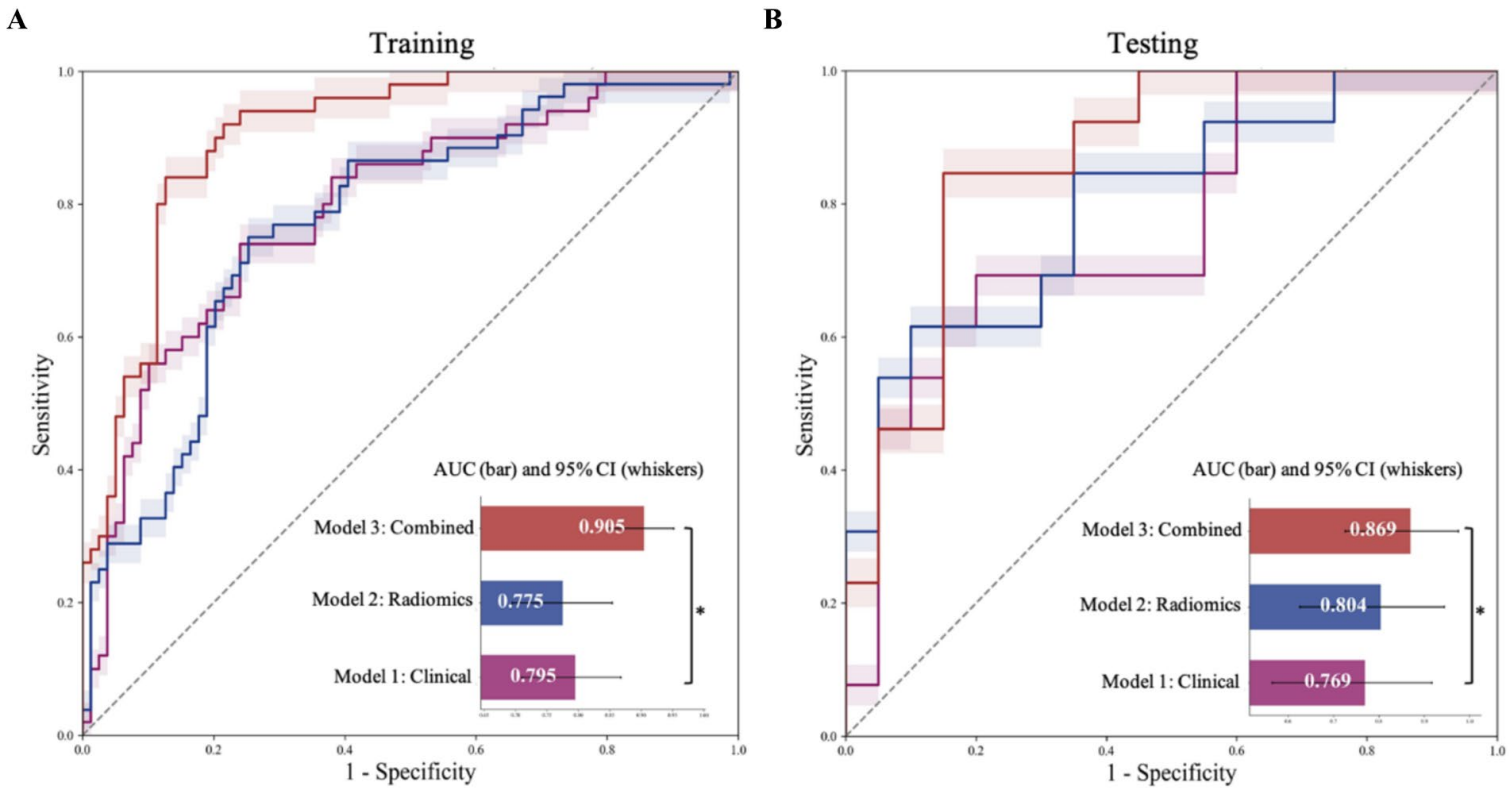
Evaluation of the performance results of the three models. Receiver operating characteristic (ROC) curves and corresponding area under the curve (AUC) values for diagnosing recurrent stroke in the training **(A)** and test **(B)** cohorts. Horizontal bars represent the median AUC, and whiskers denote the 95% confidence intervals (CIs). Pairwise comparisons between models are illustrated by vertical brackets, with asterisks indicating statistically significant differences. PVAT, perivascular adipose tissue.

This study utilized five commonly employed ML models to develop radiomics-based prediction models, clinical prediction models, and combined prediction models. This approach aimed to assess the stability of feature predictions within the ML models and to identify the optimal model for prediction. The cutoff values and predictive performances of these models in the training set were presented in Supplementary Table S7, and their performances in the testing set were shown in Table 2. The AUC values indicated that the SVM model was the most effective predictive model. The combined model, based on the decision tree, achieved the highest predictive performance (AUC = 0.87; 95% CI: 0.74–0.97), which was significantly superior to the radiomics model (AUC = 0.80; 95% CI: 0.63–0.94) and the clinical model (AUC = 0.77; 95% CI: 0.58–0.91; all *p* < 0.05). Figure 3 provided detailed ROC comparison information.

**Table 2:** The performance of different models in the testing cohort.

Model	Machine learning algorithm	Cutoff value	AUC (%, 95 CI)	Sensitivity (%, 95 CI)	Specificity (%, 95 CI)	Accuracy (%, 95 CI)
Model 1: clinical model	Decision tree	0.441	0.78 (0.59–0.93)	69.2 (41.7–92.3)	65.0 (43.5–85.0)	66.7 (48.5–81.8)
	LR	0.533	0.74 (0.56–0.90)	69.2 (43.7–92.3)	60.0 (38.9–80.0)	63.6 (48.5–78.8)
	RF	0.467	0.73 (0.53–0.90)	69.2 (41.7–92.3)	65.0 (43.5–85.0)	66.7 (48.5–81.8)
	SVM	0.403	0.77 (0.58–0.91)	69.2 (43.7–92.3)	60.0 (38.9–80.0)	63.6 (48.5–78.8)
	XGBoost	0.394	0.75 (0.55–0.90)	61.5 (35.7–87.5)	75.0 (54.5–94.1)	69.7 (54.5–84.8)
Model 2: radiomics model (PVAT + Plaque)	Decision Tree	0.479	0.76 (0.59–0.91)	69.2 (42.9–92.9)	60.0 (38.9–81.8)	63.6 (45.5–78.8)
	LR	0.456	0.80 (0.63–0.92)	76.9 (50.0–100.0)	65.0 (42.1–85.0)	69.7 (54.5–84.8)
	RF	0.51	0.73 (0.55–0.89)	53.8 (26.7–81.8)	80.0 (61.1–95.2)	69.7 (54.5–84.8)
	SVM	0.625	0.80 (0.63–0.94)	61.5 (33.3–87.5)	70.0 (47.8–90.0)	66.7 (48.5–81.8)
	XGBoost	0.332	0.79 (0.63–0.92)	69.2 (42.9–92.9)	65.0 (44.0–85.7)	66.7 (51.5–81.8)
Model 3: combined model	Decision tree	0.490	0.82 (0.67–0.94)	76.9 (53.8–100.0)	65.0 (44.4–85.7)	69.7 (54.5–84.8)
	LR	0.532	0.83 (0.69–0.96)	69.2 (41.7–92.9)	75.0 (53.8–93.3)	72.7 (57.6–87.9)
	RF	0.485	0.84 (0.69–0.98)	76.9 (50.0–100.0)	75.0 (55.0–94.1)	75.8 (60.6–87.9)
	SVM	0.702	0.87 (0.74–0.97)	84.6 (62.5–100.0)	70.0 (47.4–88.9)	75.8 (60.6–87.9)
	XGBoost	0.391	0.79 (0.61–0.93)	53.8 (27.2–80.0)	70.0 (50.0–89.5)	63.6 (48.5–78.8)

PVAT, perivascular adipose tissue; LR, logistic regression; RF, random forests; SVM, support vector machines; XGBoost, extreme gradient boosting; AUC, area under the curve; CI, confidence interval.

### 3.4 SHAP analysis of the random Forest combined model

**Figure 4:**
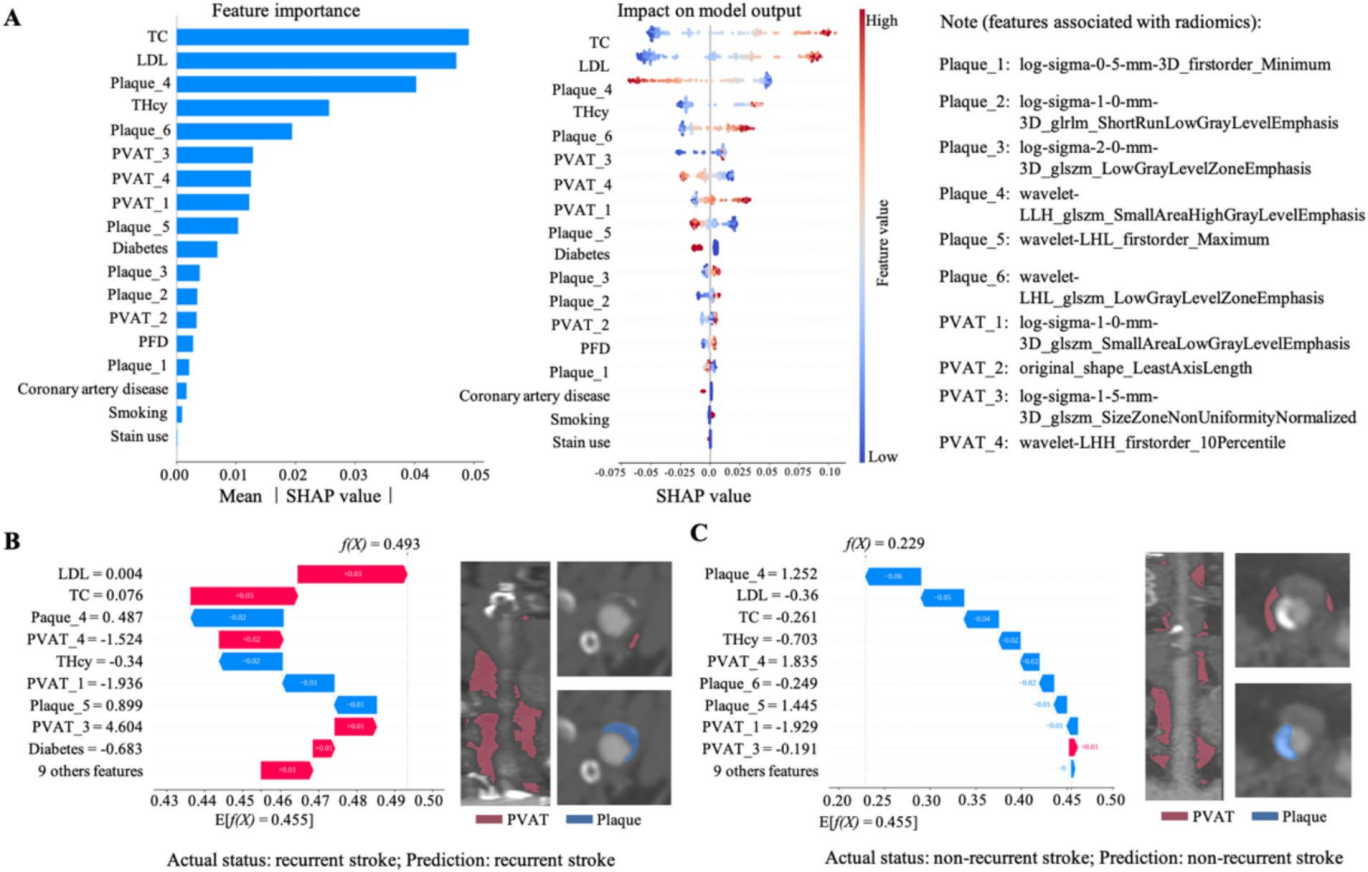
Interpretability analysis of the predictive model for recurrent stroke using SHAP (SHapley Additive exPlanations). **(A)** Bar plot showing the mean absolute SHA*p* values of top features, indicating their global importance in the model (left). Beeswarm plot illustrating the impact of each feature on model output (SHAp value), colored by feature magnitude (middle). Radiomics-associated features are detailed in the right panel. **(B,C)** Local explanation examples for two individual patients via force plots. Each bar represents the contribution of a specific feature to the final prediction score *f*(*X*). Red bars indicate positive contributions (increasing risk of recurrent stroke), blue bars indicate negative contributions. The baseline expectation E[*f*(*X*)] = 0.455 is shown at the bottom. **(B)** Patient (actual: recurrent stroke; predicted: recurrent stroke) has a higher risk score (*f*(*X*) = 0.493). **(C)** Patient (actual: non-recurrent; predicted: non-recurrent) has a lower score (*f*(*X*) = 0.229). Corresponding CT angiography images highlight segmented regions. LDL, low-density lipoprotein; PVAT, perivascular adipose tissue; TC, total cholesterol; tHcy, serum homocysteine.

To improve model interpretability, we calculated SHAP values for recurrence stroke prediction in the combined model’s SVM framework. A SHAP bar plot (Figure 4A) presents the feature importance rankings in the combined model, showing that plaque radiomic features had a stronger influence than PVAT features in predicting recurrence stroke in CAS patients. Figure 4A also shows the corresponding feature effects and the SHAP heatmap that illustrates the direction and magnitude of each feature’s impact across model. The SHAP heatmap suggests that higher values of elevated LDL, total cholesterol levels, wavelet-LHL_glszm_LowGrayLevelZoneEmphasis (radiomics feature: PVAT_6), and log-sigma-0-5-mm-3D_firstorder_Minimum (radiomics feature: PVAT_1) were all associated with a higher probability of recurrence stroke prediction. A history of diabetes was also linked to an increased risk of recurrence stroke events. Furthermore, we used visual methods to explain model predictions at the patient level. The E[f(x)] represents the baseline model prediction value and f(x) indicates the final predicted probability. Local model interpretability for two representative male patients was illustrated in Figures 4B,C.

## 4 Discussion

In this study, we developed and validated a fully automated CTA-based dual-compartment radiomics model integrating carotid plaque and perivascular adipose tissue (PVAT) features to predict ipsilateral stroke recurrence in patients with carotid atherosclerosis. The combined model demonstrated superior discriminative performance compared with clinical variables alone and radiomics-only models. Importantly, SHAP analysis revealed biologically interpretable feature contributions, with plaque texture features exerting the strong influence while PVAT-derived features provided complementary information. These findings suggest that quantitative characterization of both plaque structure and the perivascular inflammatory microenvironment provides complementary prognostic information.

Despite advances in antiplatelet therapy, statins, and blood pressure control, recurrent ischemic stroke remains a substantial clinical burden in patients with CAS. Current decision-making largely relies on luminal stenosis severity; however, stenosis alone incompletely captures plaque instability and fails to fully reflect the biological drivers of recurrent events (45, 46). Indeed, patients with similar degrees of stenosis may exhibit markedly different recurrence risks (46), highlighting the need for more refined imaging-based stratification tools. Our findings support the concept that recurrence risk is not solely determined by luminal narrowing but rather by a combination of structural plaque vulnerability and inflammatory activity reflected by the PVAT.

Radiomic features extracted from carotid plaque contributed substantially to model performance. Texture-based descriptors likely capture spatial heterogeneity, necrotic core distribution, fibrous cap disruption, and microcalcification patterns that are not readily discernible by visual assessment. Prior studies have demonstrated associations between intraplaque hemorrhage, lipid-rich necrotic core, and subsequent cerebrovascular events (8–10, 14, 15). However, most investigations have relied on qualitative assessment or focused on cross-sectional symptom status rather than longitudinal recurrence risk. By leveraging high-dimensional quantitative features, our model extends beyond conventional plaque characterization and provides individualized recurrence risk estimation. The prominence of texture features in SHAP analysis further supports the biological relevance of plaque heterogeneity as a driver of recurrent ischemic events.

Beyond plaque morphology, PVAT-derived radiomics features provided incremental predictive value. Increasing evidence suggests that PVAT functions as an active regulator of vascular inflammation through bidirectional signaling with the arterial wall. Prior studies have primarily focused on either plaque characteristics or PVAT features in isolation, and most relied on manual or semi-quantitative assessments (19, 27–31, 47). In contrast, our study extends this framework by applying three-dimensional radiomics within a fully automated pipeline to quantitatively characterize PVAT heterogeneity surrounding plaques. The added predictive value observed in the combined model suggests that the inflammatory microenvironment captured by PVAT imaging may modulate plaque destabilization and recurrent stroke risk. Importantly, SHAP analysis revealed that PVAT features contributed complementary rather than redundant information relative to plaque radiomics, supporting a dual-compartment risk framework.

Most previous ML studies in carotid disease have focused on distinguishing symptomatic from asymptomatic plaques at a single time point (19–23). While useful, such cross-sectional classification does not directly inform secondary prevention strategies. In contrast, our study specifically addressed the clinically relevant question of recurrent stroke prediction following an index ischemic event. By shifting the analytical focus from symptom identification to future event forecasting, this work aligns more closely with real-world decision-making, where clinicians must determine the intensity of medical therapy, surveillance frequency, and potential need for revascularization (48). The model’s performance in the independent testing cohort suggests that quantitative CTA phenotyping may help identify patients at particularly high recurrence risk despite standard therapy. In clinical practice, such risk stratification may support individualized secondary prevention, including closer surveillance or optimized medical therapy for high-risk patients. The imaging biomarkers may also complement traditional risk factors and luminal stenosis in identifying patients who may require further evaluation for carotid revascularization.

Several limitations should be acknowledged. First, this was a single-center retrospective study with a relatively modest sample size, which may limit generalizability. Although we employed LASSO feature selection and 10-fold cross-validation to reduce overfitting risk, the combination of a high-dimensional radiomics feature space and only 63 recurrent events may still increase the potential for model overfitting. Future studies with larger, multicenter cohorts are warranted to validate the robustness and generalizability of our findings. Second, although voxel resampling and intensity normalization were applied to mitigate variability, formal reproducibility assessment of radiomics features (e.g., test–retest or ICC analysis) was not performed. Future studies should incorporate such analyses to further validate the robustness of radiomics features across imaging conditions. Third, follow-up duration was finite, and longer-term recurrence risk beyond the study period was not assessed. In addition, we focused on binary recurrence prediction rather than time-to-event modeling; incorporating survival analysis approaches may further refine individualized risk estimation. Finally, although most patients received standard secondary prevention therapies such as antiplatelet agents, statins, and risk-factor management, treatment strategies may have varied during follow-up in routine clinical practice. These variations in medical therapy may have influenced recurrence risk but were not explicitly modeled in the present analysis.

In conclusion, a fully automated CTA-based dual-compartment radiomics model integrating carotid plaque and PVAT features improves prediction of ipsilateral stroke recurrence compared with clinical assessment alone. Quantitative characterization of both structural plaque vulnerability and the surrounding inflammatory microenvironment provides complementary prognostic information. This approach may enhance individualized risk stratification and support more precise secondary prevention strategies in patients with carotid atherosclerosis.

## Data Availability

The data that support the findings of this study are not publicly available due to privacy or ethical restrictions, but are available on request from the corresponding author.
