# Supplementary material for: CT angiography–derived plaque and perivascular fat radiomics for predicting ipsilateral stroke recurrence in patients with carotid atherosclerosis
Source: Front Neurol. 2026 Apr 16;17:1821860. doi: 10.3389/fneur.2026.1821860 (PMC13128431; doi:10.3389/fneur.2026.1821860)
Supplement: Supplementary file 1 [file Supplementary_file_1.DOCX]

Supplementary Material

# Appendix E1

The clinical characteristics collected data include: age, gender, body mass index, history of antiplatelet medication (Bayer aspirin/Plavix, Bayer AG/Sanofi), statin use (Lipitor, Pfizer Inc.), diabetes, hypertension, smoking, atrial fibrillation, dyslipidemia, coronary artery disease, history of acute cerebral infarction, triglycerides (TG), total cholesterol (TC), high-density lipoprotein cholesterol (HDL-C), low-density lipoprotein cholesterol (LDL-C), and serum homocysteine levels (tHcy). And clinical laboratory characteristics definition was as follows:

(1) Diabetes mellitus: hemoglobin A1c > 6.5% or taking diabetic medications. (2) Hypertension: blood pressure > 140/90 or on anti-hypertensive medicines. (3) Smoking history: defined as consumption of $\geq$ 1 cigarette daily for at least 3 months, unless quit over 2 years. (4) Dyslipidemia: Laboratory indicators of blood lipids include total TC, LDL, TG, and HDL, dyslipidemia is defined as TC $\geq$5.2 mmol/L, LDL $\geq$ 3.12 mmol/L, TG $\geq$ 1.7 mmol/L, HDL < 1.45 mmol/L.

# Supplementary Tables and Figures

## Supplementary Tables

**Supplementary Table S1.** CT Scanning Parameters.

| Scanner | Tube voltage (kv) | Tube current (mAs) | Detector collimation  (mm) | FOV (mm) | Pitch | Slice interval  (mm) | Slice thickness (mm) |
| --- | --- | --- | --- | --- | --- | --- | --- |
| Brilliance ICT, Philips | 100 | auto | 80 | 345×  345 | 0.725 | 0.45 | 0.9 |
| Somatom Sensation, Siemens | 150 | auto | 58 | 250×  250 | 0.7 | 5 | 1 |
| UIH | 100 | auto | 80 | 230×  230 | 0.864 | 5 | 1 |
| GE MEDICAL SYSTEMSCT | 100-120 | auto | 40 | 250×  250 | 0.984 | 0.625 | 0.625 |

**Table S2.** MRI Scanning Parameters

| Scanner | Images | Time repetition | Time echo | Slice thickness | FOV (mm) |
| --- | --- | --- | --- | --- | --- |
| Somatom Sensation, Siemens | T2-weighted images | 8500 | 94 | 5 | 256×135 |
|  | T1-weighted images | 220 | 2.77 | 5 | 226×250 |
|  | Diffusion-weighted imaging | 5400 | 94 | 6 | 229×229 |
| Brilliance ICT, Philips | T2-weighted images | 7000 | 120 | 5 | 376×171 |
|  | T1-weighted images | 300 | 2.3 | 5 | 272×218 |
|  | Diffusion-weighted imaging | 1972.74 | 65.04 | 6 | 160×123 |

**Table S3.** Baseline characteristics in the training set.

| Variables | Total (n=129) | Non-recurrent (n=79) | Recurrent (n=50) | Statistic | *P* value |
| --- | --- | --- | --- | --- | --- |
| Demographics |  |  |  |  |  |
| Age, year | 69.60 ± 7.37 | 69.29 ± 6.75 | 70.10 ± 8.31 | t=-0.61 | 0.546 |
| Sex, n (%) |  |  |  | χ²=0.82 | 0.366 |
| Female | 23 (17.83) | 16 (20.25) | 7 (14.00) |  |  |
| Male | 106 (82.17) | 63 (79.75) | 43 (86.00) |  |  |
| BMI, kg/m2 | 23.77 ± 3.38 | 24.24 ± 2.93 | 23.08 ± 3.90 | t=1.67 | 0.098 |
| Smoking, n (%) | 27 (20.93) | 13 (16.46) | 14 (28.00) | χ²=2.47 | 0.116 |
| Diabetes, n (%) | 36 (27.91) | 28 (35.44) | 8 (16.00) | χ²=5.75 | 0.016 |
| Hypertension, n (%) | 92 (71.32) | 60 (75.95) | 32 (64.00) | χ²=2.14 | 0.144 |
| Atrial fibrillation, n (%) | 7 (5.43) | 4 (5.06) | 3 (6.00) | χ²=0.00 | 1 |
| Hyperlipidemia, n (%) | 5 (3.88) | 3 (3.80) | 2 (4.00) | χ²=0.00 | 1 |
| Coronary artery disease, n (%) | 5 (3.88) | 3 (3.80) | 2 (4.00) | χ²=0.00 | 1 |
| Antiplatelet use, n (%) | 23 (17.83) | 19 (24.05) | 4 (8.00) | χ²=5.38 | 0.02 |
| Statin use, n (%) | 8 (6.20) | 7 (8.86) | 1 (2.00) | χ²=1.44 | 0.23 |
| Antihypertension use, n (%) | 12 (9.30) | 12 (15.19) | 0 (0.00) | χ²=6.67 | 0.01 |
| Antidiabetic use, n (%) | 40 (31.01) | 25 (31.65) | 15 (30.00) | χ²=0.04 | 0.844 |
| Imaging parameters |  |  |  |  |  |
| Stenosis, n (%) |  |  |  | χ²=0.72 | 0.697 |
| <50% | 10 (7.75) | 5 (6.33) | 5 (10.00) |  |  |
| 50~69% | 15 (11.63) | 10 (12.66) | 5 (10.00) |  |  |
| 70~99% | 104 (80.62) | 64 (81.01) | 40 (80.00) |  |  |
| PFD, HU | -59.92 ± 10.55 | -61.03 ± 10.48 | -58.15 ± 10.53 | t=-1.52 | 0.131 |
| Laboratory tests, mean ± SD | | | | | |
| TC | 4.29 ± 1.28 | 3.95 ± 1.05 | 4.81 ± 1.44 | t=-3.86 | <.001 |
| TG (mmol/L) | 1.44 ± 0.78 | 1.40 ± 0.81 | 1.49 ± 0.74 | t=-0.67 | 0.506 |
| HDL (mmol/L) | 1.09 ± 0.26 | 1.11 ± 0.24 | 1.07 ± 0.29 | t=0.83 | 0.406 |
| LDL | 2.64 ± 1.61 | 2.43 ± 1.87 | 2.96 ± 1.03 | t=-1.79 | 0.077 |
| THcy (μmol/L) | 14.25 ± 7.18 | 14.09 ± 8.89 | 14.43 ± 4.77 | t=-0.23 | 0.816 |
| Manufacturer, n (%) |  |  |  |  |  |
| GE | 36 (27.91) | 25 (31.65) | 11 (22.00) |  |  |
| Philips | 34 (26.36) | 23 (29.11) | 11 (22.00) |  |  |
| SIEMENS | 36 (27.91) | 30 (37.97) | 6 (12.00) |  |  |
| UIH | 23 (17.83) | 1 (1.27) | 22 (44.00) |  |  |

Note. Data are means ± standard deviations (SD) for continuous variables and frequencies and percentages for categorical variables. t = t-test; χ² = Chi-square test. BMI = body mass index, HDL = high-density lipoprotein, LDL = low-density lipoprotein, PFD = perivascular fat density, TC = total cholesterol, TG = triglycerides, tHcy = serum homocysteine.

**Table S4.** Baseline characteristics in the testing set.

| Variables | Total (n=33) | Non-recurrent (n=20) | Recurrent (n=13) | Statistic | *P* value |
| --- | --- | --- | --- | --- | --- |
| Demographics |  |  |  |  |  |
| Age, year | 68.03 ± 11.26 | 68.50 ± 8.33 | 67.31 ± 15.07 | t=0.29 | 0.772 |
| Sex, n (%) | 30 (90.91) | 17 (85.00) | 13 (100.00) | - | 0.261 |
| Female | 3 (9.09) | 3 (15.00) | 0 (0.00) |  |  |
| Male | 30 (90.91) | 17 (85.00) | 13 (100.00) |  |  |
| BMI, kg/m2 | 23.92 ± 2.59 | 23.99 ± 2.99 | 23.82 ± 2.07 | t=0.16 | 0.873 |
| Smoking, n (%) | 11 (33.33) | 5 (25.00) | 6 (46.15) | - | 0.27 |
| Diabetes, n (%) | 16 (48.48) | 10 (50.00) | 6 (46.15) | - | 1 |
| Hypertension, n (%) | 20 (60.61) | 10 (50.00) | 10 (76.92) | - | 0.159 |
| Atrial fibrillation, n (%) | 1 (3.03) | 0 (0.00) | 1 (7.69) | - | 1 |
| Hyperlipidemia, n (%) | 0 (0.00) | 0 (0.00) | 0 (0.00) | - | 1 |
| Coronary artery disease, n (%) | 2 (6.06) | 1 (5.00) | 1 (7.69) | - | 1 |
| Antiplatelet use, n (%) | 2 (6.06) | 1 (5.00) | 1 (7.69) | - | 1 |
| Statin use, n (%) | 4 (12.12) | 3 (15.00) | 1 (7.69) | - | 1 |
| Antihypertension use, n (%) | 9 (27.27) | 6 (30.00) | 3 (23.08) | - | 1 |
| Antidiabetic use, n (%) | 12 (36.36) | 6 (30.00) | 6 (46.15) | - | 0.465 |
| Imaging parameters |  |  |  |  |  |
| Stenosis, n (%) |  |  |  | - | 1 |
| <50% | 1 (3.03) | 1 (5.00) | 0 (0.00) |  |  |
| 50~69% | 4 (12.12) | 2 (10.00) | 2 (15.38) |  |  |
| 70~99% | 28 (84.85) | 17 (85.00) | 11 (84.62) |  |  |
| PFD, HU | -60.44 ± 10.74 | -60.77 ± 11.79 | -59.94 ± 9.34 | t=-0.21 | 0.832 |
| Laboratory tests, mean ± SD | | | | | |
| TC | 4.40 ± 1.59 | 3.80 ± 1.04 | 5.29 ± 1.87 | t=-2.82 | 0.009 |
| TG (mmol/L) | 1.41 ± 1.17 | 1.31 ± 1.36 | 1.57 ± 0.86 | t=-0.59 | 0.558 |
| HDL (mmol/L) | 1.03 ± 0.27 | 1.01 ± 0.29 | 1.06 ± 0.25 | t=-0.48 | 0.635 |
| LDL | 2.73 ± 1.37 | 2.22 ± 0.81 | 3.49 ± 1.69 | t=-2.42 | 0.029 |
| THcy (μmol/L) | 15.83 ± 6.31 | 11.11 ± 3.56 | 20.18 ± 5.02 | t=-5.17 | <0.001 |
| Manufacturer, n (%) |  |  |  | - | <.001 |
| GE | 4 (12.12) | 0 (0.00) | 4 (30.77) |  |  |
| Philips | 12 (36.36) | 9 (45.00) | 3 (23.08) |  |  |
| SIEMENS | 11 (33.33) | 10 (50.00) | 1 (7.69) |  |  |
| UIH | 6 (18.18) | 1 (5.00) | 5 (38.46) |  |  |

Note. Data are means ± standard deviations (SD) for continuous variables and frequencies and percentages for categorical variables. t = t-test; χ² = Chi-square test. BMI = body mass index, HDL = high-density lipoprotein, LDL = low-density lipoprotein, PFD = perivascular fat density, TC = total cholesterol, TG = triglycerides, tHcy = serum homocysteine.

**Table S5.** List of the radiomic features extracted on CTA (n=1070).

| **imageType** |
| --- |
| Original |
| LoG：sigma: [0.5, 1.0, 1.5, 2.0] |
| Wavelet |
| **featureClass** |
| **Shape (n=17)**  VoxelVolume; MeshVolume; SurfaceArea; SurfaceVolumeRatio; Compactness1; Compactness2; Sphericity; SphericalDisproportion; Maximum3DDiameter; Maximum2DDiameterSlice; Maximum2DDiameterColumn; Maximum2DDiameterRow; MajorAxisLength; MinorAxisLength; LeastAxisLength; Elongation;Flatness |
| **Firstorder (18)**  Energy; TotalEnergy; Entropy; Minimum; 10Percentile; 90Percentile; Maximum; Mean; Median; InterquartileRange; Range; MeanAbsoluteDeviation; RobustMeanAbsoluteDeviation; RootMeanSquared ; Skewness;Kurtosis; Variance; Uniformity |
| **GLCM (23)**  Autocorrelation;JointAverage;ClusterProminence;ClusterShade;ClusterTendency;Contrast;Correlation；DifferenceAverage;DifferenceEntropy;DifferenceVariance;JointEnergy;JointEntropy;Imc1;Imc2;Id;Idn;Idm;Idmn;InverseVariance;MaximumProbability;SumAverage;SumEntropy;SumSquares |
| **GLRLM (12)**  ShortRunEmphasis; LongRunEmphasis; GrayLevelNonUniformity; RunLengthNonUniformity; RunLengthNonUniformityNormalized; RunPercentage; LowGrayLevelRunEmphasis; HighGrayLevelRunEmphasis; ShortRunLowGrayLevelEmphasis; ShortRunHighGrayLevelEmphasis; LongRunLowGrayLevelEmphasis; LongRunHighGrayLevelEmphasis |
| **GLSZM (12)**  SmallAreaEmphasis; LargeAreaEmphasis; GrayLevelNonUniformity; SizeZoneNonUniformity; SizeZoneNonUniformityNormalized; ZonePercentage; LowGrayLevelZoneEmphasis; HighGrayLevelZoneEmphasis; SmallAreaLowGrayLevelEmphasis; SmallAreaHighGrayLevelEmphasis; LargeAreaLowGrayLevelEmphasis; LargeAreaHighGrayLevelEmphasis |
| **GLDM (11)**  SmallDependenceEmphasis; LargeDependenceEmphasis; GrayLevelNonUniformity; DependenceNonUniformity; DependenceNonUniformityNormalized; DependenceEntropy; DependenceVariance; SmallDependenceLowGrayLevelEmphasis; SmallDependenceHighGrayLevelEmphasis; LargeDependenceLowGrayLevelEmphasis; LargeDependenceHighGrayLevelEmphasis |
| **NGTDM (5)**  Coarseness; Contrast; Busyness; Complexity; Strength |

**Table S6.** Selected radiomics feature of pericarotid adipose tissue (PVAT) and plaque

| **PVAT radiomics features** |
| --- |
| PVAT_rad1: log-sigma-1-0-mm-3D_glszm_SmallAreaLowGrayLevelEmphasis |
| PVAT_rad2: original_shape_LeastAxisLength |
| PVAT_rad3: log-sigma-1-5-mm-3D_glszm_SizeZoneNonUniformityNormalized |
| PVAT_rad4: wavelet-LHH_firstorder_10Percentile |
| **Plaque radiomics features** |
| Plaque_rad1: log-sigma-0-5-mm-3D_firstorder_Minimum |
| Plaque_rad2: log-sigma-1-0-mm-3D_glrlm_ShortRunLowGrayLevelEmphasis |
| Plaque_rad3: log-sigma-2-0-mm-3D_glszm_LowGrayLevelZoneEmphasis |
| Plaque_rad4: wavelet-LLH_glszm_SmallAreaHighGrayLevelEmphasis |
| Plaque_rad5: wavelet-LHL_firstorder_Maximum |
| Plaque_rad6: wavelet-LHL_glszm_LowGrayLevelZoneEmphasis |

**Table S7**. The performance of different models in the training and the testing cohort.

| **Model** | **ML Algorithm** | **Set** | **Cutoff** | **AUC (95% CI)** | **Accuracy (95% CI)** | **Sensitivity (95% CI)** | **Specificity (95% CI)** | **PPV (95% CI)** | **NPV (95% CI)** |
| --- | --- | --- | --- | --- | --- | --- | --- | --- | --- |
| Model3: Combined model | Desicion Tree | Train | 0.49 | 0.900 (0.842-0.951) | 0.860 (0.798-0.915) | 0.860 (0.761-0.943) | 0.861 (0.779-0.935) | 0.796 (0.685-0.900) | 0.907 (0.835-0.962) |
|  | Desicion Tree | Test | 0.49 | 0.815 (0.669-0.938) | 0.697 (0.545-0.848) | 0.769 (0.538-1.000) | 0.650 (0.444-0.857) | 0.588 (0.350-0.824) | 0.812 (0.625-1.000) |
|  | LR | Train | 0.532 | 0.873 (0.812-0.929) | 0.837 (0.767-0.899) | 0.840 (0.735-0.939) | 0.835 (0.747-0.914) | 0.764 (0.641-0.867) | 0.892 (0.822-0.959) |
|  | LR | Test | 0.532 | 0.831 (0.686-0.959) | 0.727 (0.576-0.879) | 0.692 (0.417-0.929) | 0.750 (0.538-0.933) | 0.643 (0.385-0.883) | 0.789 (0.579-0.950) |
|  | RF | Train | 0.485 | 0.912 (0.857-0.955) | 0.845 (0.767-0.907) | 0.900 (0.811-0.979) | 0.810 (0.714-0.895) | 0.750 (0.638-0.859) | 0.928 (0.861-0.985) |
|  | RF | Test | 0.485 | 0.842 (0.688-0.976) | 0.758 (0.606-0.879) | 0.769 (0.500-1.000) | 0.750 (0.550-0.941) | 0.667 (0.400-0.909) | 0.833 (0.647-1.000) |
|  | SVM | Train | 0.702 | 0.905 (0.844-0.952) | 0.860 (0.791-0.915) | 0.840 (0.729-0.936) | 0.873 (0.797-0.943) | 0.808 (0.690-0.913) | 0.896 (0.824-0.958) |
|  | SVM | Test | 0.702 | 0.869 (0.738-0.967) | 0.758 (0.606-0.879) | 0.846 (0.625-1.000) | 0.700 (0.474-0.889) | 0.647 (0.412-0.867) | 0.875 (0.700-1.000) |
|  | XGB | Train | 0.391 | 0.865 (0.799-0.924) | 0.814 (0.744-0.884) | 0.720 (0.596-0.837) | 0.873 (0.793-0.941) | 0.783 (0.660-0.897) | 0.831 (0.750-0.904) |
|  | XGB | Test | 0.391 | 0.788 (0.613-0.925) | 0.636 (0.485-0.788) | 0.538 (0.272-0.800) | 0.700 (0.500-0.895) | 0.538 (0.250-0.834) | 0.700 (0.500-0.895) |
| Model 2: Radiomics model  (PVAT + Plaque) | Desicion Tree | Train | 0.479 | 0.831 (0.756-0.896) | 0.763 (0.687-0.832) | 0.846 (0.745-0.932) | 0.709 (0.603-0.812) | 0.657 (0.543-0.767) | 0.875 (0.781-0.946) |
|  | Desicion Tree | Test | 0.479 | 0.762 (0.590-0.909) | 0.636 (0.455-0.788) | 0.692 (0.429-0.929) | 0.600 (0.389-0.818) | 0.529 (0.294-0.765) | 0.750 (0.533-0.938) |
|  | LR | Train | 0.456 | 0.794 (0.715-0.865) | 0.725 (0.641-0.802) | 0.865 (0.769-0.951) | 0.633 (0.527-0.738) | 0.608 (0.493-0.714) | 0.877 (0.789-0.956) |
|  | LR | Test | 0.456 | 0.796 (0.631-0.923) | 0.697 (0.545-0.848) | 0.769 (0.500-1.000) | 0.650 (0.421-0.850) | 0.588 (0.333-0.812) | 0.812 (0.600-1.000) |
|  | RF | Train | 0.51 | 0.779 (0.694-0.851) | 0.740 (0.664-0.809) | 0.635 (0.508-0.756) | 0.810 (0.725-0.895) | 0.688 (0.550-0.812) | 0.771 (0.674-0.847) |
|  | RF | Test | 0.51 | 0.727 (0.552-0.889) | 0.697 (0.545-0.848) | 0.538 (0.267-0.818) | 0.800 (0.611-0.952) | 0.636 (0.333-0.909) | 0.727 (0.526-0.909) |
|  | SVM | Train | 0.625 | 0.775 (0.686-0.849) | 0.748 (0.672-0.817) | 0.750 (0.636-0.860) | 0.747 (0.640-0.843) | 0.661 (0.540-0.778) | 0.819 (0.727-0.897) |
|  | SVM | Test | 0.625 | 0.804 (0.632-0.939) | 0.667 (0.485-0.818) | 0.615 (0.333-0.875) | 0.700 (0.478-0.900) | 0.571 (0.308-0.846) | 0.737 (0.526-0.929) |
|  | XGB | Train | 0.332 | 0.874 (0.809-0.931) | 0.779 (0.710-0.847) | 0.885 (0.793-0.964) | 0.709 (0.600-0.810) | 0.667 (0.556-0.778) | 0.903 (0.823-0.969) |
|  | XGB | Test | 0.332 | 0.788 (0.626-0.922) | 0.667 (0.515-0.818) | 0.692 (0.429-0.929) | 0.650 (0.440-0.857) | 0.562 (0.318-0.800) | 0.765 (0.556-0.941) |
| Model 1: Clinical model | Desicion Tree | Train | 0.441 | 0.785 (0.698-0.860) | 0.721 (0.636-0.798) | 0.660 (0.512-0.789) | 0.759 (0.667-0.845) | 0.635 (0.500-0.768) | 0.779 (0.684-0.868) |
|  | Desicion Tree | Test | 0.441 | 0.779 (0.590-0.926) | 0.667 (0.485-0.818) | 0.692 (0.417-0.923) | 0.650 (0.435-0.850) | 0.562 (0.312-0.800) | 0.765 (0.556-0.941) |
|  | LR | Train | 0.533 | 0.810 (0.730-0.884) | 0.791 (0.713-0.860) | 0.720 (0.595-0.840) | 0.835 (0.746-0.912) | 0.735 (0.611-0.857) | 0.825 (0.734-0.904) |
|  | LR | Test | 0.533 | 0.742 (0.555-0.902) | 0.636 (0.485-0.788) | 0.692 (0.437-0.923) | 0.600 (0.389-0.800) | 0.529 (0.278-0.737) | 0.750 (0.500-0.941) |
|  | RF | Train | 0.467 | 0.766 (0.673-0.850) | 0.729 (0.651-0.799) | 0.660 (0.512-0.789) | 0.772 (0.683-0.857) | 0.647 (0.517-0.780) | 0.782 (0.685-0.870) |
|  | RF | Test | 0.467 | 0.733 (0.530-0.900) | 0.667 (0.485-0.818) | 0.692 (0.417-0.923) | 0.650 (0.435-0.850) | 0.562 (0.312-0.800) | 0.765 (0.556-0.941) |
|  | SVM | Train | 0.403 | 0.795 (0.709-0.872) | 0.752 (0.674-0.822) | 0.740 (0.610-0.857) | 0.759 (0.662-0.854) | 0.661 (0.544-0.788) | 0.822 (0.721-0.905) |
|  | SVM | Test | 0.403 | 0.769 (0.581-0.914) | 0.636 (0.485-0.788) | 0.692 (0.437-0.923) | 0.600 (0.389-0.800) | 0.529 (0.278-0.737) | 0.750 (0.500-0.941) |
|  | XGB | Train | 0.394 | 0.747 (0.651-0.827) | 0.752 (0.674-0.822) | 0.600 (0.444-0.745) | 0.848 (0.764-0.924) | 0.714 (0.583-0.844) | 0.770 (0.678-0.859) |
|  | XGB | Test | 0.394 | 0.748 (0.548-0.898) | 0.697 (0.545-0.848) | 0.615 (0.357-0.875) | 0.750 (0.545-0.941) | 0.615 (0.357-0.867) | 0.750 (0.550-0.929) |

Note. PVAT, perivascular adipose tissue; BDT, Bagging_Desicion_Tree; LR, logistic regression; RF, random forests; SVM, support vector machines; XGBoost, extreme gradient boosting; AUC, area under the curve; CI, confidence interval; PPV, Positive predictive value; NPV, negative predictive value.

## Supplementary Figures

**
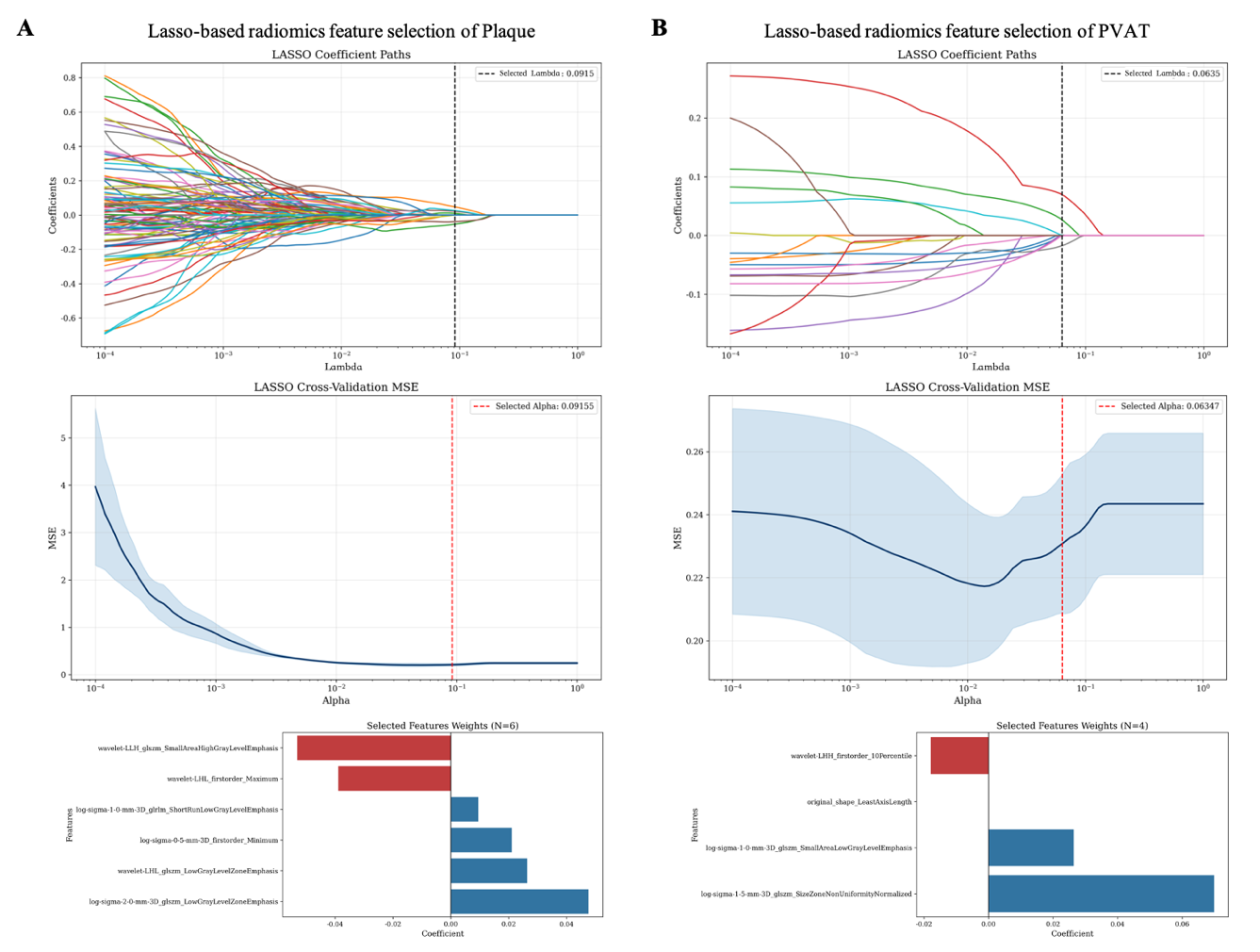
**

**Supplementary Figure S1.** **LASSO-based radiomics feature selection for plaque (a) and perivascular adipose tissue (PVAT) (b) regions.** Top panel shows LASSO coefficient paths as a function of the regularization parameter λ, with each line representing one radiomic feature; vertical dashed line indicates the optimal λ selected via cross-validation. Middle panel displays mean squared error (MSE) from 10-fold cross-validation across α values, with shaded area indicating standard deviation; red dashed line marks the λ minimizing MSE. Bottom panel presents bar plot of coefficients for the selected features after LASSO shrinkage — positive coefficients (blue) indicate risk-increasing features, negative coefficients (red) indicate protective or inverse associations.
